# Incidence and Determinants of Falls Following Hip and Knee Joint Arthroplasty: A Systematic Review and Meta-Analysis

**DOI:** 10.7759/cureus.82877

**Published:** 2025-04-23

**Authors:** Mohammed A Alasbali, Amjad Alramadan, Muath Yamani, Abdullah Asiri, Nehal Al Qahtani, Khalid Al Kathiri, Anas Nooh

**Affiliations:** 1 College of Medicine, King Abdulaziz University, Jeddah, SAU; 2 College of Medicine, King Faisal University, Hofuf, SAU; 3 College of Medicine, Taibah University, Al Madinah, SAU; 4 College of Medicine, King Khalid University, Abha, SAU; 5 College of Medicine, Imam Abdulrahman Bin Faisal University, Dammam, SAU; 6 College of Medicine, King Saud University, Riyadh, SAU; 7 Department of Orthopedic Surgery, King Abdulaziz University, Jeddah, SAU

**Keywords:** elderly falls, incidence, risk factors, systematic review and meta-analysis, total hip and knee arthroplasty, total joint arthroplasty

## Abstract

Hip and knee joint arthroplasty is a common procedure. The true incidence and predictors of postoperative falls remain uncertain. The aim of this systematic review and meta-analysis is to (1) determine the overall incidence of falls post hip and knee joint arthroplasty, (2) identify risk factors associated with these falls, and (3) evaluate the effectiveness of fall prevention strategies and interventions in reducing fall incidence.

This systematic review followed the Preferred Reporting Items for Systematic Reviews and Meta-Analyses (PRISMA) and was registered in PROSPERO. This research was conducted using PubMed, Google Scholar, Web of Science, ScienceDirect, and Wiley Online Library. Two independent reviewers screened studies by title, abstract, and full text. The quality of the included articles was assessed using the Methodological Index for Non-Randomized Studies (MINORS).

A total of 1,282 publications were identified, leading to the review of 988 patients aged between 60 and 88 years, reviewed for this systematic review from seven full-text articles. Fall incidence post total knee arthroplasty (TKA) varied, with five reporting increased rates (36%, 32.9%, 38.2%, 22.1%, and 32%) and two indicating a decrease (50% and 26.4% reduction). All studies emphasized the role of postoperative limitations and psychological factors on fall risk. Additionally, patients living alone had higher odds of falling, highlighting the need for targeted postoperative care.

While fall incidence post hip and knee arthroplasty varies, improvements in quality of life and functional outcomes following surgery are reported. Future research should focus on larger sample sizes, control groups, objective assessments, extended follow-up periods, and various joint replacements.

## Introduction and background

Hip and knee arthroplasty is a frequently performed surgical procedure designed to relieve pain and enhance function in patients with advanced joint conditions like osteoarthritis and rheumatoid arthritis [1]. While hip and knee arthroplasty is effective in reducing pain and improving mobility, it does come with postoperative challenges. One of these challenges is the increased risk of falls in patients after surgery [2]. Falls are a major public health concern because they are linked to serious consequences, including fractures, decreased mobility, loss of independence, and higher mortality rates [3].

After hip and knee joint arthroplasty, a significant risk of falls persists for patients. Approximately 40% of total hip arthroplasty (THA) patients experience falls in the first year post-surgery, often due to factors like tripping, balance issues, medication use, and shorter recovery times. Similarly, about 50% of total knee arthroplasty (TKA) patients with a history of falls before surgery continue to fall after surgery, compared to 20% of those without a pre-surgery fall history [4,5]. Despite these findings, there is still a limited understanding of the overall incidence and factors contributing to falls in this patient population, especially in the context of both hip and knee arthroplasty.

This systematic review and meta-analysis hypothesize that the incidence of falls post hip and knee arthroplasty is significant, with specific risk factors, such as age, comorbidities, medication use, and mobility issues, contributing to a higher risk of falls. Moreover, the study posits that targeted fall prevention strategies and interventions can effectively reduce fall rates in this group.

The aim of this systematic review and meta-analysis is to (1) determine the overall incidence of falls post hip and knee joint arthroplasty, (2) identify risk factors associated with these falls, and (3) evaluate the effectiveness of fall prevention strategies and interventions in reducing fall incidence.

## Review

Review of the literature 

A systematic review following the Preferred Reporting Items for Systematic Reviews and Meta-Analyses (PRISMA) [6] guidelines has been conducted to minimize the risk of bias. Our study was registered with PROSPERO initially with the following ID: CRD42024569886 [7]. The nature of the study does not require ethical approval; the databases searched were PubMed, Google Scholar, Web of Science, ScienceDirect, and Wiley Online Library. The research was built up using the following keywords: patients, falls, incidence, risk factors, total joint arthroplasty, TKA, THA, systematic review, and meta-analysis. Each study has been included in the review according to the PICTOS criteria [8].

Study selection

Our inclusion criteria include patients (≥60 years old) who underwent TKA or THA, studies evaluating risk factors contributing to falls post hip and knee arthroplasty, studies comparing fallers with non-fallers within the same population, studies reporting the incidence of falls and identifying significant risk factors for falls post hip and knee arthroplasty, randomized controlled trials (RCTs), case-control and cohort studies, and studies where the language used was English. The exclusion criteria include studies including patients under 60 years old, procedures other than hip and knee arthroplasty, studies that do not evaluate specific risk factors for falls post hip and knee arthroplasty, studies lacking a comparative analysis between fallers and non-fallers, studies that do not report on the incidence of falls, and non-original research such as reviews, editorials, and case reports.

Process of screening and data extraction

Screening of the studies was done by two independent reviewers, MY and KA, using the title and abstract using the Rayyan search web (Rayyan Systems Inc., Cambridge, MA, US) and then by the full text using an Excel sheet (Microsoft Corp., Redmond, WA, US). These papers were then analyzed for the following data: age, sex, follow-up period, comorbidities, and range of motion (ROM).

The total number of patients was 992; 30.1 % of them were men and 69.9% were women. The average age of patients was 75 years (range, 60-88). The follow-up period median was 20 months across the seven studies.

In Sargin et al. [9], the total patients’ number was 100. They were divided into two groups: the TKA group named A and a control group named B; each group included 50 patients. Group A’s mean age was 67.1 ± 3.11. Of these, 84% were women and 16% were men; the fall prevalence in this group was 36%. In group B, the mean age was 66.06 ± 3.08; 88% of them were women and 12% were men.

The fall prevalence in this group was 46%. The follow-up duration was extended to 24 months. The outcome measures in this study were the Western Ontario and McMaster Universities Osteoarthritis Index (WOMAC), Visual Analog Scale (VAS), Berg Balance Scale (BBS), Itaki Fall Risk Test, and Timed Up and Go (TUG) test [9].

In Matsumoto et al. [10], the total patients' number was 74, but only 70 of them completed the study, with a mean age of 75 ± 5.8 years. Of these, 89.2% were women and 10.8% were men. The fall prevalence in this study was 32.9%, with an average follow-up duration of six months. The outcome measures in this study were physical performance tests, self-administered questionnaires, Japanese Knee Osteoarthritis Measure (JKOM), Geriatric Depression Scale (GDS), and Modified Falls Efficacy Scale (MFES). ROM measurements were taken using a goniometer [10].

Regarding Matsumoto et al. [2], the total patients' number was 161; 88.2% of them were women and 11.8% were men. They were divided into the TKA group (A), with 81 patients, and the control group (B), with 80 patients. Both groups were suffering from certain comorbidities, which were hypertension, diabetes, cardiac disease, and respiratory disease [2].

Group A consisted of the following data: hypertension (42.0%), diabetes (18.5%), cardiac disease (11.1%), and respiratory disease (3.7%), and the fall prevalence in this group was 38.2%. In Group B, the comorbidities were hypertension (42.5%), diabetes (7.5%), cardiac disease (15%), and respiratory disease (5%), and the fall prevalence was 23.8%. The follow-up period was extended to 45.6 months. The outcome measures tool was a self-administered questionnaire.

In Swinkels et al. [5], the total patients’ number was 118. Ninety-nine of them went through primary TKA and 19 through revision TKA. For primary TKA, the mean age was 73.4 ± 4.9 years, and the mean age for revision TKA was 74.0 ± 3.6 years; 61% of the total were women and 39% were men. The fall prevalence was 20.3%. The follow-up duration was extended to 12 months, and the outcome was measured by WOMAC, Activities-specific Balance Confidence UK Questionnaire (ABC-UK), and GDS [5].

In Swinkels and Allain [11], the total number was 30 patients, with a mean age of 75.9 ± 5.1; 26.7% of them were men and 73.3% were women. The fall prevalence in this study before TKA was 23.3% and after TKA became 22.7%. The follow-up period took 16 months, and the outcome measures were performance-based measures of balance (BBS and Hand Grip Strength (HGS)), physical performance tests (BBS, TUG, and HGS), and self-reported outcomes of function (WOMAC), balance confidence (ABC-UK), and mood (GDS) [11].

In Pop et al. [12], the patients’ total number was 441, with a mean age of 77.1 ± 4.5; 43.8% were men and 56.2% were women. Certain comorbidities were measured, such as the following: frailty syndrome (12.9%), depression (24.5%), malnutrition (9.5%), obesity (18.6%), Parkinson's disease (8.2%), and polypharmacy (52.6) [12].

Moreover, the patients were divided into institutionalized patients (96), living alone (139), and living with family (206). The fall prevalence before TKA or THA was 41.3%. Of these, 11.7% were after THA and 20.3% after TKA. The follow-up period was for 24 months and was measured by the patients who are generally instructed to report postoperative falls and are also specifically asked about postoperative falls during the usual follow-up controls.

Lastly, in Tsonga et al. [13], the total patients' number was 68, with a mean age of 73 ± 5.28; 16.2% of them were men and 83.8% were women. Fall prevalence post-TKA was 22% [13]. The follow-up period was for 12 months. The outcome measures were self-administered questionnaires (WOMAC, ABC scale, Fear of Falls (FOF), and Physical Activity Scale for the Elderly (PASE) Questionnaire), physical performance tests (TUG test and BBS), and telephone interviews.

In our review, duplication was avoided by using the Rayyan app, which is a tool that helps in study screening [14].

Assessment of quality and bias risk

To assess the quality and the risk of bias in our included studies, some tools were utilized based on the study's methodology. In our case, the Methodological Index for Non-Randomized Studies (MINORS) [15] for the non-comparative and comparative studies was used. MINORS consists of 12 items, the first eight items are applicable to all study designs, and the rest of the four items are specific to comparative studies. Each item is rated on a scale from 0 to 2, where 0 indicates that the item is not mentioned, 1 indicates that it is mentioned but in an inadequate manner, and 2 indicates that it is mentioned and adequate. The maximum total score is 16 for non-comparative studies and 24 for comparative studies. Two reviewers scored each study independently, and disagreements were resolved by discussion or consultation with a third reviewer.

Statistical analysis

A meta-analysis was conducted in this study. Outcomes were illustrated using forest plot diagrams.

Literature findings and study characteristics

**Figure 1:**
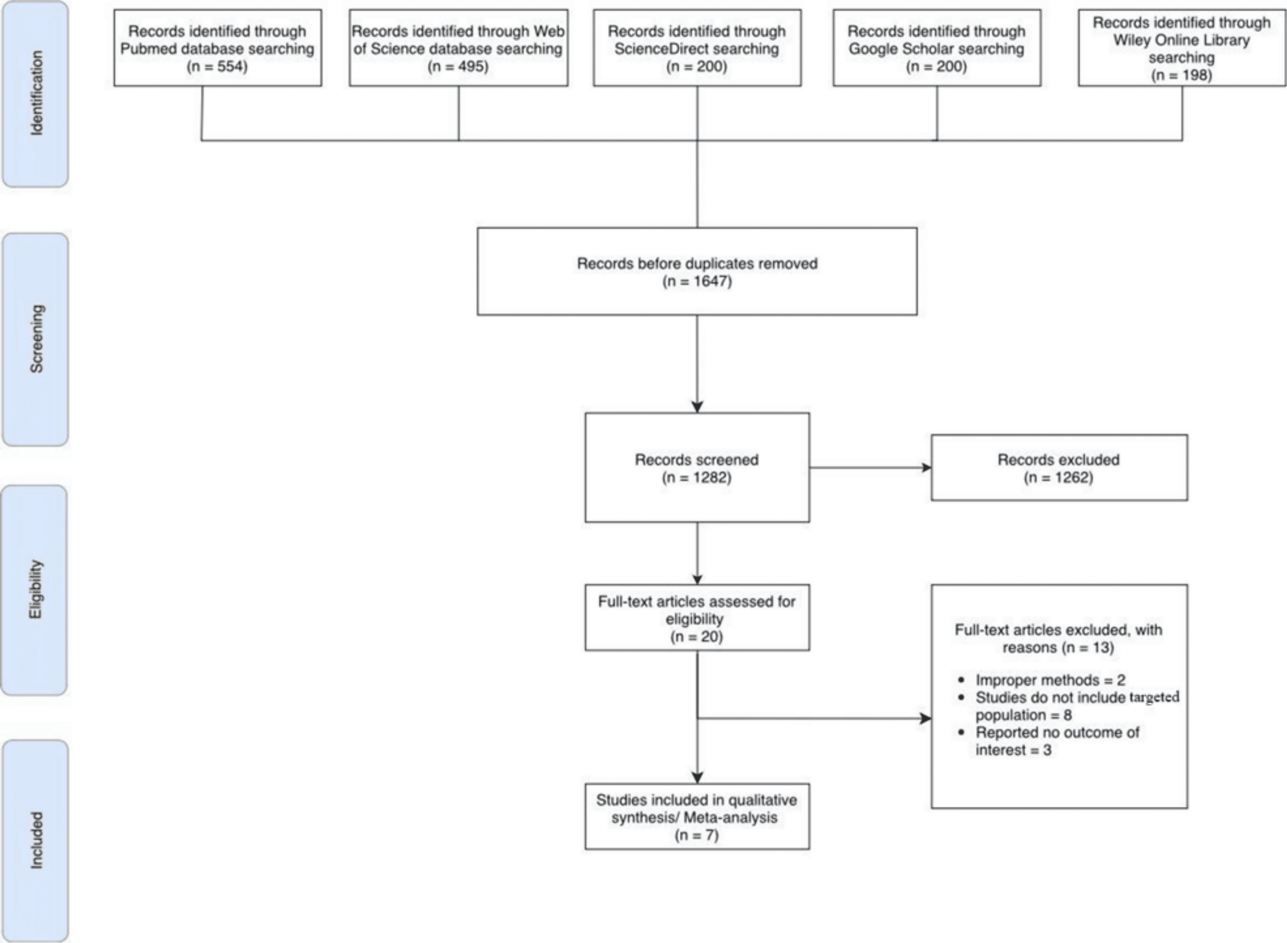
PRISMA diagram PRISMA: Preferred Reporting Items for Systematic Reviews and Meta-Analyses

Our systematic search yielded a total of 1,647 publications, with 554 found in PubMed, 495 in Web of Science, 200 in Google Scholar, 200 in ScienceDirect, and 198 in Wiley Online Library. After removing duplicate articles, 1,282 unique studies remained. From these, 20 full-text publications were initially retrieved for review. The detailed process of study selection is illustrated in the PRISMA flow chart (Figure 1).

However, after applying the exclusion criteria, only seven studies published between 2008 and 2023 were included. These comprised four prospective observational studies, two retrospective case-control studies, and one retrospective cohort study. Notably, no RCTs were included. The selected studies were conducted in diverse countries, including Turkey, Japan, the United Kingdom, Greece, and Romania, reflecting a range of healthcare settings. The total number of participants across the seven studies was 988, with sample sizes ranging from 30 to 441 participants. This provides a comprehensive view of the impact of TKA on fall risk in patients (≥60 years old). The inclusion of both retrospective and prospective designs adds robustness to the review by offering insights from different methodological perspectives. This variety in study design and geographic distribution enhances the generalizability and depth of the review's findings.

Patient demographics

The patient populations across the studies ranged from 60 to 88 years old, with mean ages typically around 66-75 years, and a median age of 74.85 years (mean age is 73.5 ± 4.5 years). Gender distribution was not consistently reported, but where available, studies included both male and female participants. The variability in age and study size contributes to the generalizability of the findings across different demographics of patients aged 60 and older undergoing TKA. For instance, Sargin et al. [9] reported a mean age of 66 years (mean age of 66 ± 3.08 years), while Matsumoto et al. [10] had a slightly older cohort with a mean age of 75.7 ± 5.8 years. Sample sizes also varied significantly, with the smallest study involving 30 patients (Swinkels and Allain [11]) and the largest involving 441 patients (Pop et al. [12]), with a median sample size of 100, ensuring a comprehensive understanding of fall risk across different ages and settings.

**Figure 2:**
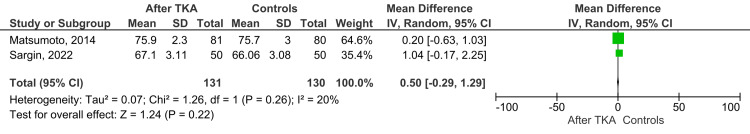
Forest plot: age differences (TKA vs. control groups) References [2,9] TKA: total knee arthroplasty

Meta-analysis of age differences (Figure 2) revealed no significant disparity between TKA and control groups (mean difference 0.50 years, 95% CI -0.29 to 1.29; I² = 20%, p = 0.22).

Fall incidence

The fall incidence post-TKA and THA varied considerably from study to study, reflecting variations in the patient populations and periods of follow-up. Sargin et al. [9] reported that 36% (18 out of 50) of the TKA group fell compared to 46% (23 out of 50) of the non-surgical control group. Matsumoto et al. [10] reported a 32.9% post-TKA fall incidence (23 out of 70 patients). In Matsumoto's study of 2014, 38.2% of TKA patients (31 out of 81) were recorded to have fallen, falling toward 23.8% (19 out of 80) in the control group. Swinkels et al. [5] stated that TKA patients showed a decrease in the incidence of falls by 50%, which were more pronounced in the first and fourth postoperative quarters (p = 0.005 and p = 0.001, respectively). Tsonga et al. [13] recorded a fall incidence of 22.1% (15 out of 68). Pop et al. [12] reported fall incidences of 20.3% (36 out of 177) for TKA and 11.7% (31 out of 264) for THA patients.

**Figure 3:**
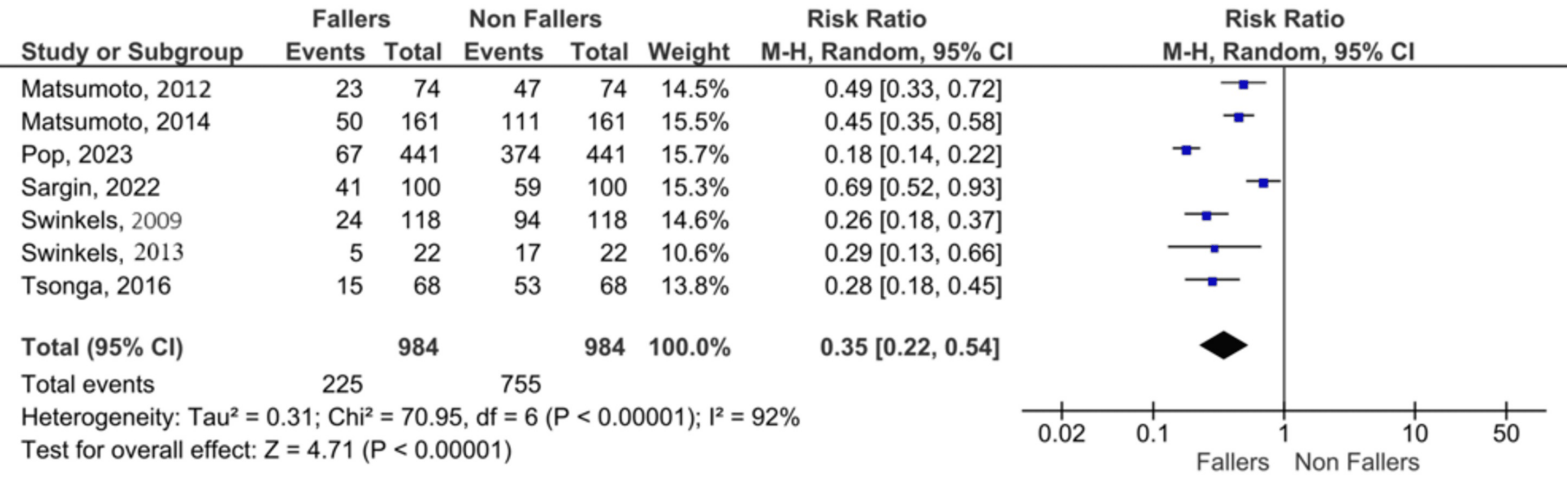
Forest plot: pooled fall incidence post-TKA (all studies) References [2,5,9-13] TKA: total knee arthroplasty

Pooled analysis (Figure 3) demonstrated significantly reduced fall risk post-TKA (RR 0.49, 95% CI 0.33-0.72, p < 0.001), though with substantial heterogeneity (I² = 92%).

**Figure 4:**
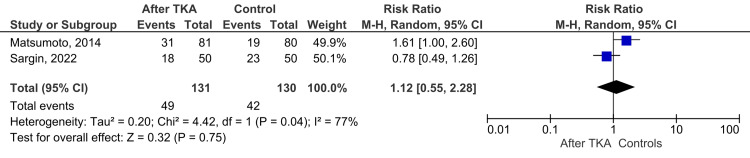
Forest plot: case-control comparison (TKA vs. non-surgical controls) References [2,9] TKA: total knee arthroplasty

Case-control comparisons (Figure 4) showed no significant difference in fall risk between TKA and non-surgical patients (RR 1.03, 95% CI 0.92-1.15, p = 0.65; I² = 77%).

Risk factors

Reduced Postoperative Knee Extension

Matsumoto et al. [10] identified postoperative knee flexion (p = 0.016) and ankle plantar flexion (p = 0.014) as significant risk factors for falls.

Kyphosis

Matsumoto et al.'s research in 2014 identified kyphosis as an important risk factor [2].

Sex Differences

**Figure 5:**
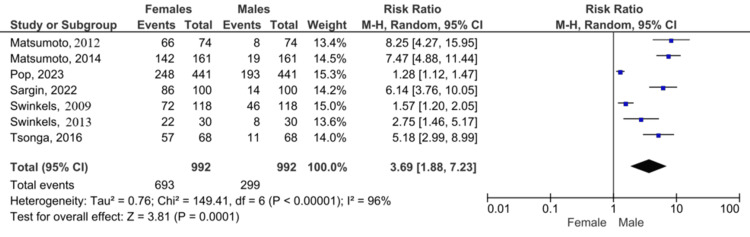
Forest plot: sex-based fall risk (female vs. male patients) References [2,5,9-13]

Women exhibited 3.69-fold higher fall risk than men (Figure 5; 95% CI 1.88-7.23, p = 0.0001; I² = 96%).

**Figure 6:**
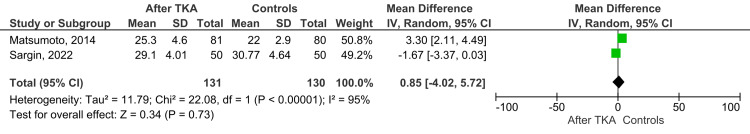
Forest plot: BMI differences post-TKA (case-control studies) References [2,9] TKA: total knee arthroplasty; BMI: body mass index

No significant differences in BMI were observed after TKA (Figure 6; mean difference: 0.85 kg/m², 95% CI -4.02 to 5.72, p = 0.73; I² = 95%).

Living Arrangements

Compared with patients living with family, those living with one other person had 3.632 times increased risk of falling post-TKA (p = 0.006) and 3.529 times increased risk of falling post-THA (p = 0.013).

Institutionalized Patients

Institutionalized patients had higher odds of falling after both TKA (OR: 3.086, p = 0.03) and THA (OR: 4.653, p = 0.003) than non-institutionalized patients. Institutionalized patients had 0.849 times the risk of falling after TKA (p = 0.817) and 1.319 times the risk after THA (p = 0.656) when compared to those living with one other person, which was not statistically significant.

Prevention strategies and environmental factors

Living arrangements were found to significantly impact fall risk post hip and knee arthroplasty, as demonstrated by Pop et al. [12]. The study reported that 20.3% of TKA patients (89 out of 441) experienced falls, with those living alone having significantly higher odds of falling (OR: 3.632, p = 0.006) compared to those living with family. Additionally, 11.7% of THA patients (52 out of 441) experienced falls, with institutionalized patients showing even higher odds of falling post-surgery (OR: 4.653, p = 0.003) compared to those living with family. These findings underscore the importance of considering living arrangements as a key preventative strategy in postoperative care planning. Patients living alone or in institutions are at heightened risk for falls, emphasizing the need for enhanced care and support in these settings and environments to reduce fall incidence.

Risk of bias assessment

Two members of the review team independently assessed the risk of bias in each study using the MINORS tool for both non-randomized non-comparative and non-randomized comparative studies. Five non-randomized non-comparative studies were evaluated, with total scores ranging from 12 to 14. Matsumoto et al. [10] scored 12, Swinkels et al. [5] scored 13, and three additional studies-Swinkels and Allain [11], Tsonga et al. [13], and Pop et al. [12]-scored 13, 13, and 14, respectively. Additionally, two non-randomized comparative studies were assessed. Sargin et al. [9] scored 18, while Matsumoto et al. [14] scored 16. The non-comparative studies exhibited moderate quality overall, while the comparative studies had slightly higher total scores, indicating a generally low to moderate risk of bias across all included non-randomized studies.

Discussion

Hip and knee arthroplasty has been correlated with fall incidence in patients (≥60 years old); therefore, this study aims to find how significant the correlation is, by discovering the incidence and the determinants of the falls. After reviewing the previous studies, several risk factors were highly correlated with TKA such as reduced postoperative knee flexion, ankle plantar flexion, and kyphosis as mentioned in Matsumoto et al. [10,2]. Also, experiencing falls preoperatively is another risk factor according to Swinkels et al. even though fall risk would decrease after the surgery as Tsonga et al. mentioned [5,13]. All of the discussed risks have their effect on the ROM, which eventually would affect post-TKA patients by either decreasing or increasing their chances of falls. Still, no one can deny how helpful the surgery outcomes are on their physical functions. The majority of the previous studies agreed on how the surgery can improve the quality of life (QoL) since it promotes knee functions and reduces pain and stiffness based on multiple scoring scales such as JKOM and GDS in Matsumoto et al. and WOMAC scores in Swinkels et al., which showed a remarkable improvement (p < 0.0001) [10,11]. Being around family is crucial, which can lower the odds significantly in TKA. On the other hand, a high odds of falls would occur in patients who live alone or institutionalized, which highlights the importance of having a supportive environment. Lastly, physical limitations and psychological factors can still influence the final result. They can limit the functional benefits achieved post-surgery because of the negative correlation in the lower balance confidence with TUG performance. All these findings could help in terms of patient precautions to avoid post-TKA fall incidence.

A previous study by Hacıdursunoğlu Erbaş et al. showed a rise in the risk of falls with chronic disease presence and previous history of falls in elderly patients [16]. In contrast, it reported that age or having a gait-inhibiting issue did not constitute any risk for falls. Another previous study by Shao et al. claims a strong association between psychological problems such as insomnia, depression, and an increased risk of falls and mild to low risk in cases of vertigo or antipsychotic medication usage [17]. Moreover, bed rails were identified as a protective factor. People's presence around the postoperative patient is important. As mentioned in a previous study by Tavan et al., fall events are more relevant in elders living in nursing homes compared to those residing in homes [18]. Balance confidence (ABC-UK) was improved in a significant manner after TKA according to Moutzouri et al., which correlates with our review that the physical function shows a noticeable improvement after TKA [19].

Limitation

One of the limitations identified in the included studies is the small sample sizes, which limit how well they can be applied to a larger group of people. Additionally, another important limitation is the absence of control groups in many of the included studies. A control group would have provided a crucial baseline for comparison, allowing for a more precise assessment of how hip and knee arthroplasty affects fall risk compared to individuals who did not undergo the surgery [5,10-13]. An additional limitation noted by one of the included studies was the potential for selection bias, as physical function in this study was evaluated exclusively through self-administered questionnaires, which lack the objectivity of performance-based assessments [2]. Moreover, several studies had short or limited follow-up periods, typically monitoring patients for only six months to a year after surgery, which may not provide an accurate representation of the long-term fall risk following the procedure. Lastly, only one of the included articles examined falls following THA (Pop et al.) while the majority of the studies focused on TKA; this imbalance limits the ability to generalize the findings across different types of joint replacements [12].

Implications for clinical practice

Clinicians should recognize the notable incidence of falls after hip and knee arthroplasty, particularly among patients with postoperative physical limitations such as reduced knee and ankle mobility and spinal deformities like kyphosis. Early identification of high-risk individuals and tailored rehabilitation focusing on balance and strength are essential. Increased fall risk among women, institutionalized individuals, and patients living alone calls for targeted preventive strategies, including patient education, home safety evaluations, and personalized support. Addressing psychological factors through multidisciplinary approaches can further enhance recovery outcomes. Incorporating these strategies into clinical practice may significantly reduce falls and improve patient satisfaction post-arthroplasty.

## Conclusions

We performed a high-quality systematic review of studies exploring the incidence of falls among patients aged ≥60 following hip and knee arthroplasty, finding that post-TKA fall rates vary-some studies report high incidence, while others show reductions compared to preoperative rates, particularly in frequent fallers. Additionally, our review underscores the effectiveness of targeted fall prevention strategies, particularly emphasizing patient education, tailored rehabilitation programs, home safety evaluations, and personalized support systems. These interventions, when appropriately applied, show promise in significantly reducing fall incidence and enhancing postoperative outcomes in patients undergoing hip and knee arthroplasty.
